# High-Density Lipoprotein-Associated miR-223 Is Altered after Diet-Induced Weight Loss in Overweight and Obese Males

**DOI:** 10.1371/journal.pone.0151061

**Published:** 2016-03-10

**Authors:** Fatiha Tabet, Luisa F. Cuesta Torres, Kwok Leung Ong, Sudichhya Shrestha, Sébastien A. Choteau, Philip J. Barter, Peter Clifton, Kerry-Anne Rye

**Affiliations:** 1 Lipid Research Group, School of Medical Sciences, University of New South Wales Australia, Randwick, New South Wales, Australia; 2 School of Pharmacy and Medical Sciences, Division of Health Sciences, University of South Australia, Adelaide, South Australia, Australia; University of Catanzaro Magna Graecia, ITALY

## Abstract

**Background and Aims:**

microRNAs (miRNAs) are small, endogenous non-coding RNAs that regulate metabolic processes, including obesity. The levels of circulating miRNAs are affected by metabolic changes in obesity, as well as in diet-induced weight loss. Circulating miRNAs are transported by high-density lipoproteins (HDL) but the regulation of HDL-associated miRNAs after diet-induced weight loss has not been studied. We aim to determine if HDL-associated miR-16, miR-17, miR-126, miR-222 and miR-223 levels are altered by diet-induced weight loss in overweight and obese males.

**Methods:**

HDL were isolated from 47 subjects following 12 weeks weight loss comparing a high protein diet (HP, 30% of energy) with a normal protein diet (NP, 20% of energy). HDL-associated miRNAs (miR-16, miR-17, miR-126, miR-222 and miR-223) at baseline and after 12 weeks of weight loss were quantified by TaqMan miRNA assays. HDL particle sizes were determined by non-denaturing polyacrylamide gradient gel electrophoresis. Serum concentrations of human HDL constituents were measured immunoturbidometrically or enzymatically.

**Results:**

miR-16, miR-17, miR-126, miR-222 and miR-223 were present on HDL from overweight and obese subjects at baseline and after 12 weeks of the HP and NP weight loss diets. The HP diet induced a significant decrease in HDL-associated miR-223 levels (p = 0.015), which positively correlated with changes in body weight (r = 0.488, p = 0.032). Changes in miR-223 levels were not associated to changes in HDL composition or size.

**Conclusion:**

HDL-associated miR-223 levels are significantly decreased after HP diet-induced weight loss in overweight and obese males. This is the first study reporting changes in HDL-associated miRNA levels with diet-induced weight loss.

## Introduction

A recent study from our laboratory showed that both a long-term low-fat high protein (HP) diet and a low-fat high carbohydrate (HC) diet reduce body weight in overweight and obese males[1]. However, the consumption of the HP diet was more effective at improving body composition. Participants who consumed the HP diet lost less lean mass compared with the participants who received the HC diet[1]. Due to our limited understanding of the molecular mechanisms that link dietary intervention, particularly HP and HC diets, to weight loss, research into these homeostatic mechanisms has enormous potential to produce meaningful advances in the identification of novel drug targets and therapeutic strategies for weight loss.

MicroRNAs (miRNAs) are small non-coding RNAs that suppress gene expression through post-transcriptional regulation[2, 3]. Plasma and serum miRNAs exist in a free form, bound to protein complexes, contained within exosomes and microvesicles or associated with HDL[4–6]. Recent work from our group has shown that HDL-associated miRNAs are dynamic and functional. They are also involved in intercellular communication[7]. Indeed, we and others have reported that miR-223 in particular is transferred between cells by HDL in a novel cell-to-cell communication network[6, 7]. However, it is currently unknown if HDL-miRNAs contribute to the metabolic responses that are apparent during weight loss in overweight and obese subjects.

Multiple miRNAs have been associated with obesity and metabolic disorders in humans. These include miR-16, miR-17, miR-126, miR-222 and miR-223[8–11]. In fact, obesity is associated with decreased levels of miR-17 in the blood, subcutaneous and omental adipose tissue from obese subjects[8], decreased levels of miR-126 in both white adipose tissue from obese women and isolated fat cells[9] and decreased levels of serum miR-223[10]. Morbidly obese individuals also have markedly increased circulating levels of miRNAs such as miR-222[11].

It has been shown that circulating miRNAs can predict weight loss in obese subjects and therefore could be used as prognostic biomarkers in response to diet. In fact, Milagro *et al*. showed that baseline expression of several miRNAs including miR-223, in peripheral blood mononuclear cells of obese women can predict the response to a hypocaloric diet[12]. Interestingly, this study showed that basal miR-223 levels were lower in the peripheral blood mononuclear cells from obese women that did not respond to a low calorie (800–880 kcal/day) diet intervention compared to obese women who successfully lost weight[12].

As there are no reports of changes in HDL-associated miRNA levels with diet-induced weight loss in the literature, we have examined the effects of weight loss on HDL levels of miR-16, miR-17, miR-126, miR-222 and miR-223, all of which have a known association with obesity and are HDL-associated[6]. To our knowledge this is the first study looking at the effects of weight loss on HDL-associated miRNAs.

## Materials and Methods

### Participants

47 men were selected from a cohort of 120 men who participated in a weight loss study contrasting a high protein (HP) diet (30% of energy) with a normal protein (NP) diet (20% of the energy). The samples were selected randomly based on sample availability (n = 27 for NP group and n = 20 for HP group) for both week 0 and week 12. The outcomes of the weight loss study are fully reported[1]. The participants provided a written consent to participate in this study and the Human Research Ethics Committee of the Commonwealth Scientific and Industrial Research Organisation (CSIRO) approved the study.

### Weight and body composition

Body weight was measured using calibrated electronic digital scales (Mercury; AMZ 14, Tokyo, Japan) and body composition (total body fat mass and fat free mass) was measured by dual-energy X-ray absorptiometry (Lunar Prodigy; General Electric, Madison, WI, USA)[1].

### Biochemical analysis

Serum lipids (total cholesterol and HDL-cholesterol), triglycerides, and plasma glucose were measured using commercial enzymatic kits (Roche Diagnostics, Basel, Switzerland) on a Hitachi 902 autoanalyzer (Roche Diagnostics, Indianapolis, IN, USA)[1]. Low-density lipoprotein (LDL) cholesterol was calculated using a modified Friedewald equation[1, 13].

### HDL isolation

HDL were isolated from 600 μl of serum by immunoprecipitation using goat anti-apoA-I beads as previously described. Briefly, human serum was applied to a column containing goat anti-human apoA-I antibody covalently coupled to Cyanogen bromide (CNBr)-activated Sepharose 4B (Amersham Pharmacia Biotech). To remove proteins non-specifically bound to the beads, the column was washed 10 times with 1X Tris Buffered Saline (TBS). HDL that bound to the column were eluted with stripping buffer (0.1 M acetic acid) and neutralized immediately with 1 M Tris, pH 11 (final concentration, 0.11 M). Samples were further concentrated using Amicon Ultra-15 centrifuge filter unit (Millipore, Catalogue # UFC901024) and Amicon Ultra-0.5, ultracel-10 membrane (Millipore, Catalogue # UFC501096).

### Serum concentration of human HDL constituents

HDL were isolated from serum using the Polyethylene Glycol 6000 (PEG-6000) method[14]. Briefly, 100 μL of the PEG-6000 (200 mg/ml) solution was added to 100 μL of human serum (1:1 v/v ratio of serum:PEG). The mixture was briefly vortexed and incubated for 20 min at 4°C to precipitate very low and low-density lipoproteins. The mixture was then centrifuged at 15,000 rpm for 20 minutes. The supernatant was collected and the serum concentration of apolipoprotein (apo) A-I and apoA-II were determined immunoturbidometrically. The serum concentration of total cholesterol (TC), unesterified cholesterol (UC); cholesterol esters (CE) and triglycerides (TG)[5] were determined enzymatically on a Beckman Coulter AU480 Auto-Analyser (Beckman Coulter, Inc.; CA, USA).

### Characterisation of HDL particles

HDL particle sizes were determined using 3/40% non-denaturing polyacrylamide gradient gel electrophoresis (GGE) and Coomassie staining, with reference to high molecular weight standards of known diameter[15].

### Mature miRNA individual TaqMan assays

HDL-miRNA levels at baseline and after 12 weeks of weight loss were assessed by real-time PCR TaqMan miRNA assays. Briefly, total RNA was isolated from HDL using Qiazol miRNAeasy kits (Qiagen) as previously described[7]. Total RNA was quantified by spectrophotometry (Nanovue). HDL-associated RNA was reversed transcribed using the TaqMan microRNA reverse transcription kit (Applied Biosystems) according to the manufacturer’s instructions. Subsequently, 7.5 μl of the reverse transcription product was used for detecting miRNA expression using TaqMan miRNA Assay Kits (Applied Biosystems) for the specific miRNAs. Values were normalized to both *Caenorhabditis elegans* (Cel) miR-39 (which was spiked into the samples after the Qiazol step) and HDL total protein concentration determined by the BCA assay (Thermo Scientific, USA). Results are expressed as 2^-(Ct(microRNA)-Ct(Cel-miR-39))^.

### Statistical analysis

Data analysis was performed using SPSS 22 (IBM, Armonk, NY). Data were examined for normality based on skewness and kurtosis before analysis. Non-normally distributed variables (fold changes of miR-222 and miR-223) were normalised prior to analysis using natural logarithmic (ln) transformation. Comparison of clinical characteristics between two independent groups at baseline was performed using student t-test. To evaluate the effect of diet treatment on clinical characteristics, two-tailed paired t-test was used to assess significant differences between baseline and after 12 weeks of treatment. As miRNA levels at baseline were highly skewed, analysis focused on the fold changes over 12 weeks, which were calculated as the ratios of the miRNA levels at 12 weeks to those at baseline, and were assessed by one-sample t-test. Multiple testing correction was performed by Bonferroni correction for the two diet treatment groups, in which the threshold of p value for significance was <0.025. Correlations between fold changes of miRNA levels and changes in clinical characteristics over 12 weeks were performed using bivariate Pearson correlation coefficients. Results for the serum concentration of human HDL constituents were analyzed by 2-way ANOVA with Tukey’s multiple comparison test. Statistical significance was set at *p*<0.05.

## Results

The 47 individuals included in this study were older than the other 73 individuals who commenced the weight loss study[1] (mean age 52.3±8.6 vs 47.7±9.3 years, p = 0.007), but there was no significant difference in body mass index. Table 1 shows the clinical characteristics of the 47 subjects at baseline as well as the changes in these characteristics from baseline to 12 weeks. At baseline, there were no significant differences in the clinical characteristics between high protein (HP) and normal protein (NP) groups, except that the HP group had significantly lower triglyceride levels compared to the NP group (*p* = 0.013). Over the 12 weeks of weight loss, body weight, total cholesterol, LDL cholesterol, triglyceride levels, fasting glucose, total fat mass, total lean mass, total body fat percentage, calculated fat mass, calculated lean mass, calculated fat free mass, total abdominal fat mass and abdominal fat percentage significantly decreased, whereas total body fat free mass percentage increased in both HP and NP groups (all *p*<0.05). When compared to the NP diet, the HP diet group showed a greater decrease in total body fat percentage and calculated fat mass, and a greater increase in total body fat free mass percentage (*p*<0.05 for all).

**Table 1:** Clinical characteristics at baseline and changes over 12 weeks of weight loss diets.

Clinical Characteristics	Baseline		Change over 12 weeks		*P value*			
	High protein (n = 20)	Normal protein (n = 27)	High protein (n = 20)	Normal protein (n = 27)	Baseline^a^	Change in High protein group^b^	Change in Normal protein group^b^	Difference in change between groups^c^
Age (years)	53.2 ± 51.7	51.7 ± 8.6	-	-	0.586	-		-
BMI (kg/m^2^)	32.7 ± 4.2	32.6 ± 4.4	-	-	0.933	-		-
Height (m)	1.75 ± 0.07	1.78 ± 0.07	-	-	0.241	-		-
Body Weight (kg)	100.5 ± 11.4	103.3 ± 15.9	-9.0 ± 4.7	-8.7 ± 4.0	0.472	<0.001	<0.001	0.803
Total cholesterol (mmol/l)	5.27 ± 0.95	5.32 ± 0.86	-0.58 ± 1.05	-0.83 ± 0.80	0.875	0.024	<0.001	0.354
HDL cholesterol (mmol/l)	1.27 ± 0.33	1.22 ± 0.33	+0.08 ± 0.22	-0.02 ± 0.20	0.588	0.118	0.647	0.121
LDL cholesterol (mmol/l)	3.28 ± 0.92	3.10 ± 0.78	-0.45 ± 0.88	-0.48 ± 0.78	0.485	0.033	0.004	0.899
Triglycerides (mmol/l)	1.62 ± 0.70	2.22 ± 0.86	-0.45 ± 0.54	-0.73 ± 0.57	0.013	0.001	<0.001	0.101
Glucose (mmol/l)	5.83 ± 0.53	6.02 ± 1.15	-0.31 ± 0.46	-0.32 ± 0.71	0.493	0.006	0.025	0.957
Total fat mass (kg)	31.2 ± 6.7	32.8 ± 7.5	-7.1 ± 3.4	-5.1 ± 3.2	0.456	<0.001	<0.001	0.052
Total lean mass (kg)	64.3 ± 5.8	65.9 ± 10.3	-1.4 ± 2.3	-3.6 ± 5.0	0.515	0.013	0.001	0.061
Total body fat (%)	32.4 ± 4.2	33.1 ± 3.9	-5.1 ± 2.9	-2.8 ± 3.5	0.588	<0.001	<0.001	0.029
Total body fat free mass (%)	67.6 ± 4.2	66.9 ± 3.9	+5.1 ± 2.9	+2.8 ± 3.5	0.588	<0.001	<0.001	0.029
Calculated fat mass (kg)	32.8 ± 7.2	34.3 ± 7.5	-7.5 ± 3.5	-5.2 ± 3.3	0.496	<0.001	<0.001	0.032
Calculated lean mass (kg)	64.3 ± 5.9	65.8 ± 10.2	-1.7 ± 3.1	-3.8 ± 5.1	0.570	0.029	<0.001	0.095
Calculated fat free mass (kg)	67.6 ± 6.0	69.0 ± 10.2	-1.6 ± 3.0	-3.6 ± 4.8	0.563	0.028	<0.001	0.099
Total abdominal fat mass (kg)	2.66 ± 0.74	2.92 ± 1.04	-0.71 ± 0.37	-0.60 ± 0.42	0.351	<0.001	<0.001	0.383
Total abdominal lean mass (kg)	3.52 ± 0.64	3.53 ± 0.93	-0.09 ± 0.25	-0.13 ± 0.56	0.958	0.131	0.255	0.757
Abdominal fat (%)	42.6 ± 5.5	44.6 ± 4.6	-7.2 ± 4.5	-4.8 ± 3.7	0.173	<0.001	<0.001	0.055

Data are presented as mean ± SD.

^a^Comparison of clinical characteristics between the two diet groups at week 0 (student t-test).

^b^Changes from baseline to week 12 within each group (two-tailed paired t-test).

^c^Difference in the changes over time between the two diet groups (student t-test).

**Fig 1:**
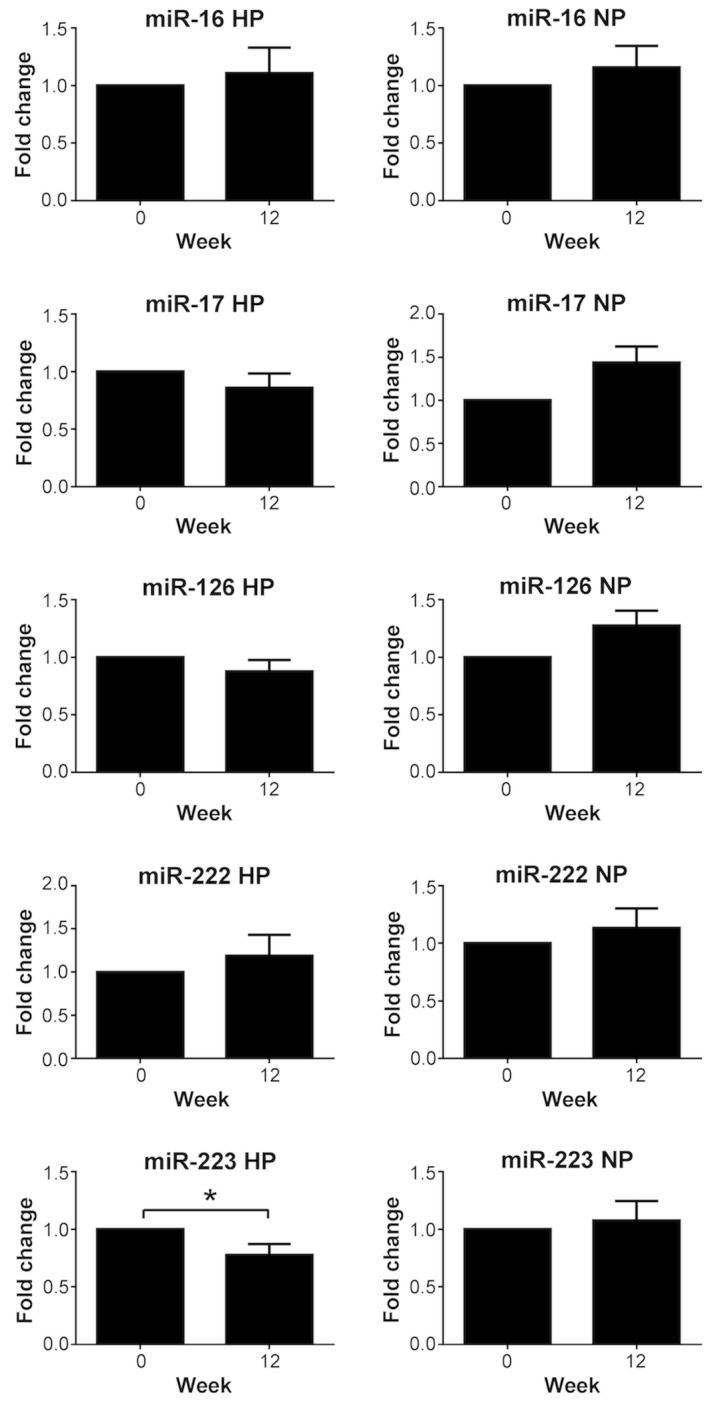
Fold-changes of HDL-miRNA levels after 12 weeks of high protein and normal protein diets. HDL-miRNAs miR-16, miR-17, miR-126, miR-222 and miR-223 were assessed by real-time PCR TaqMan miRNA assays and normalized to both Caenorhabditis elegans (Cel) miR-39 and HDL total protein concentration. Results are expressed as fold change. Values represent the mean±SEM of n = 20 for the high protein (HP) diet group and n = 27 for the normal protein (NP) diet group. *P<0.05 (after multiple testing correction).

Fig 1 and Table 2 show the fold changes of HDL-associated miR-16, miR-17, miR-126, miR-222 and miR-223 from overweight and obese subjects over 12 weeks of HP or NP weight loss diet. For miR-223, a significant decrease was found in the HP group (p = 0.015), but not in the NP group. The difference in the fold changes between the two diet groups did not reach statistical significance (p = 0.084). For miR-16, miR-17, miR-126 and miR-222, no significant change was found over 12 weeks in the HP or NP group. miR-17 and miR-126 levels increased in the NP group, but this did not reach significance after multiple testing correction.

**Table 2:** Changes of HDL-associated miR-16, miR-17, miR-126, miR-222 and miR-223 levels over 12 weeks of weight loss diet (n = 47).

HDL-miRNA	*P value*	
	Change in high protein group (n = 20)	Change in normal protein group (n = 27)
miR-16	0.633	0.406
miR-17	0.278	0.026
miR-126	0.225	0.042
miR-222*	0.522	0.237
miR-223*	**0.015**†	0.416

*Fold changes of miR-222 and miR-223 over 12 weeks were ln-transformed before analysis due to skewed distribution.

† Significant P value after multiple testing correction.

As HDL-associated miR-223 levels decreased in the HP group, correlation analysis of the changes in HDL-associated miR-223 levels with the changes in clinical characteristics over 12 weeks in the HP group was assessed (Table 3). In the HP diet group, changes in the level of HDL-associated miR-223 were positively correlated with changes in body weight over 12 weeks of diet (Table 3).

**Table 3:** Bivariate correlation of changes in HDL-associated miR-223 levels with changes in clinical characteristics over 12 weeks of weight loss diet in the high protein group (n = 20).

Change	*r*
Body Weight	0.488*
Total cholesterol	-0.101
HDL cholesterol	0.266
LDL cholesterol	-0.170
Triglycerides	-0.060
Glucose	-0.014
Total fat mass	0.416
Total lean mass	0.226
Total body fat	0.147
Total body fat free mass	-0.147
Calculated fat mass	0.452
Calculated lean mass	0.247
Calculated fat free mass	0.240
Total abdominal fat mass	0.459
Total abdominal lean mass	-0.042
Abdominal fat	0.257

Fold changes of miR-223 level were ln-transformed before analysis due to skewed distribution (*p<0.05).

**Fig 2:**
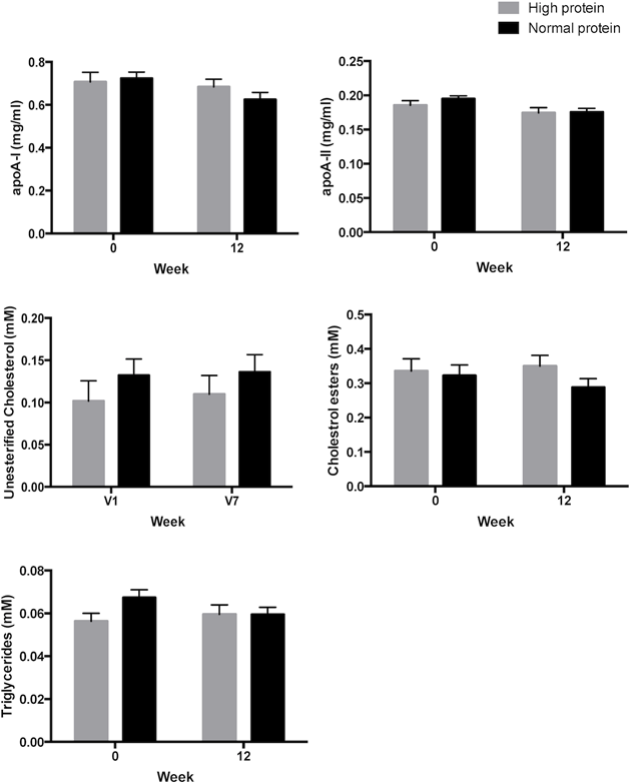
Serum concentration of human HDL constituents. Apolipoprotein A-I (apoA-I), apolipoprotein A-II (apoA-II), unesterified cholesterol, cholesterol ester and triglyceride levels. Results are presented as mean±SEM.

Serum concentration of the human HDL constituents, including apoA-I, apoA-II, UC, CE and TG did not significantly change after 12 weeks of either HP or NP diet. These results indicate that changes in HDL-miR-223 levels are not associated with changes in HDL composition (Fig 2).

**Fig 3:**
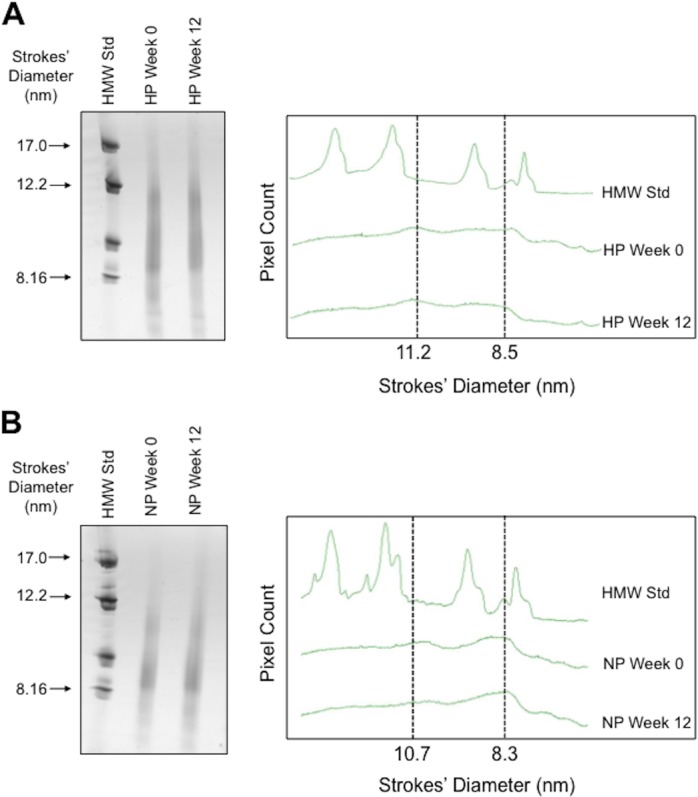
HDL particle sizes before and after 12 weeks of high protein (A) or normal protein (B) diets. HDL particle sizes were determined using 3/40% non-denaturing polyacrylamide GGE and Coomassie staining, with reference to high molecular weight standard (HMW Std) of known diameter. Scans of gradient gels of HDL isolated from representative subjects after high protein (HP) or normal protein (NP) diets are shown.

HDL isolated using goat anti-apoA-I beads were subjected to non-denaturing gradient gel electrophoresis. In the representative gels in Fig 3, the HDL consisted of two main populations of particles 8.3–8.5 and 10.7–11.2 nm in diameter. HDL particle size did not change after 12 weeks of either HP or NP diet. These results indicate that changes in HDL-associated miR-223 levels are independent of changes in HDL size.

## Discussion

It has recently been demonstrated that HDL transport multiple miRNAs, including miR-16, miR-17, miR-126, miR-222 and miR-223[6]. In this study we asked if the levels of these miRNAs are altered in HDL after diet-induced weight loss in overweight and obese males. After multiple testing corrections, we found that only the level of HDL-associated miR-223 was significantly decreased in overweight and obese male subjects after 12 weeks of high protein diet-induced weight loss. We also found that changes in HDL-miR-223 levels are not related to HDL composition or size as 12 weeks of HP diet was accompanied by a decrease in the level of HDL-associated miR-223, while the composition and size of the HDL particles did not change.

Our results suggest that, among all the HDL-associated miRNAs that were studied in this project, HDL-associated miR-223 is the most sensitive to metabolic changes after 12 weeks of diet-induced weight loss. These results are consistent with the fact that miR-223 has previously been associated with metabolic disorders such as obesity and diabetes[16–18]. The association of miR-223 with metabolic disorders has been demonstrated both *in vitro* and *in vivo*. Indeed, miR-223 regulates glucose metabolism and GLUT4 expression in neonatal rat cardiomyocytes[16], is up-regulated in insulin-resistant human hearts[16] and down-regulated in plasma from patients with type 2 diabetes[18]. In obese mice, miR-223 is overexpressed in adipocytes[17].

Circulating miR-223 levels have previously been associated with diet-induced weight loss[12]. Indeed, Milagro *et al*. have shown that peripheral blood mononuclear cells (PBMCs) miR-223 levels predict the response to diet-induced weight loss with basal levels of miR-223 being lower in people that did not respond to the weight loss intervention[12]. Contrary to our study, the study from Milagro *et al*. was performed in women, who received a hypocaloric diet (800–880 kcal/day) for 8-weeks and the results were reported for miRNAs in PBMCs[12]. This suggests that basal levels of miRNAs, including miR-223, could potentially be used as prognostic biomarkers and may forecast the response to a hypocaloric diet. Additional evidence that miR-223 is sensitive to metabolic change comes from a recent study showing that serum miR-223 levels are increased after one-hour of acute anaerobic exercise[19] and immediately after completing a marathon race[20].

Although circulating miRNAs are stable in plasma, and their levels can predict human diseases [11, 21], we and others have shown that circulating miRNAs, including HDL-associated miRNAs, are also very dynamic through their involvement in intercellular communication [6, 7, 22]. Indeed, we have previously shown that miR-223 transfers from HDL to human coronary artery endothelial cells, where it inhibits inflammation by reducing mRNA and protein levels of intercellular adhesion molecule-1[7]. Therefore, these results clearly suggest that HDL-associated miRNAs are functional and can regulate gene expression in recipient cells and therefore may contribute to intercellular communication. The results in the present study show that after 12 weeks of HP diet-induced weight loss, the levels of HDL-associated miR-223 are significantly decreased in obese and overweight individuals. These changes positively correlated with changes in body weight. This raises the possibility that HDL-associated miR-223 may play a role in the metabolic changes associated with diet-induced weight loss through its involvement in intercellular communication[16]. Further studies are required to confirm this hypothesis.

The present study also examined other HDL-associated miRNAs (miR-16, miR-17, miR-126, miR-222) that are associated with obesity and/or weight loss but they were not significantly altered with diet. The explanation for this lack of change could be related to the type of weight loss intervention. Indeed, the effects of weight loss interventions on plasma (as opposed to HDL-associated) miRNA levels suggest that different weight loss interventions (exercise, diet or surgery) may differentially affect circulating miRNAs levels[11, 12, 20, 23]. For example, circulating levels of miR-16-1 and miR-222 are decreased following gastric bypass bariatric surgery, but not affected by diet-induced weight loss[11].

Further studies in other clinical settings with larger sample size are needed to confirm the findings in the present study. Further studies targeting the role of HDL-associated miR-223 will also provide insight into the effect of dysregulated HDL-miRNAs in obesity-associated diseases and the metabolic changes associated with weight loss.
